# Mouldability of Additively Manufactured Attachments on Multipoint Tools

**DOI:** 10.3390/ma15228137

**Published:** 2022-11-16

**Authors:** Thomas Herzog, Carsten Tille, Hermann Seitz

**Affiliations:** 1 Department of Mechanical, Automotive and Aeronautical Engineering, Munich University of Applied Sciences, 80335 Munich, Germany; 2 Microfluidics, Faculty of Mechanical Engineering and Marine Technology, University of Rostock, 18059 Rostock, Germany; 3 Department Life, Light and Matter, University of Rostock, 18059 Rostock, Germany

**Keywords:** multipoint moulding with additive attachments, fused filament fabrication, multipoint moulding, carbon fibre reinforced plastics, additive manufacturing

## Abstract

Enhanced multipoint moulding with additive attachments (EMMA) is a process combining vacuum-assisted multipoint moulding (VAMM) and additively manufactured moulding attachments for carbon fibre reinforced plastics (CFRP) component production. The aim of this initial study is to investigate the mouldability of the additively manufactured attachments on the multipoint tool. For this purpose, two different test specimens were defined, the VAMM machine was adjusted, the attachments were additively built with the robot on the curved silicone interpolation layer and lastly, the CFRP specimens were moulded. The fabrication results were analysed with surface comparisons to check that there was no displacement of the attachments during moulding. A visual evaluation of the manufactured components was carried out, and the overall dimensional accuracy was assessed by comparing the surface with the target geometry. The results showed a very good agreement between the shapes before and after the moulding and thus prove that the attachments were not postponed in the moulding process. The optical evaluation confirms good moulding results for the parts manufactured with the enhanced multipoint moulding with additive attachments. Moreover, the evaluation shows that the major parts of the specimens comply with the permissible tolerance of t = 6 mm defined in ISO 20457. To the authors’ best knowledge, this is the first study that has investigated the entire EMMA process and systematically proved the mouldability of the additively manufactured attachments on multipoint tools.

## 1. Introduction

In recent years, the customer demand for individualised products has increased significantly. This leads to reduced batch sizes in production and thus to an increase of small batch production or even the production of individual items. When using fibre-reinforced plastics, the necessary moulds have to be disposed of after being used only a few times, which is long before the end of their service life. This leads to economic and ecological disadvantages due to the long production time of the mould and its premature disposal.

Vacuum-assisted multipoint moulding (VAMM) as a special type of multipoint tooling was developed to compensate for these disadvantages. The development of this technology over time has already been discussed in Herzog and Tille [1] in detail. The multipoint moulding technology, also referred to as multipoint tooling, was initially invented by Cochrane [2] for sheet metal forming with manual adjustment of the pins. Later studies focused on the automatization of the pin adjustment [3,4,5]. Moreover, there were different developments for different materials and part sizes. For example, Walczyk et al. [6] investigated the technology for CFRP parts, Wang and Yuan [7] present the forming of very large aluminium sheet material, Tan et al. [8] discuss the forming of titanium for skull reconstruction and Hagemann [9] uses the technology for injection moulding. The research in this article is based on the developments described in Wimmer [10] and Wimmer et al. [11]. In these studies, the process is adapted to the production of carbon fibre reinforced plastics (CFRP), and a test rig is set up.

The VAMM machine essentially consists of a matrix of pins that can be adjusted in height and thus create the mould. To compensate for the discrete jumps in height between the individual pins, they are covered with an interpolation layer made of silicone. For the representation of concave geometries, a vacuum is built up underneath this silicone interpolation layer. The subsequent process steps at VAMM are similar to those of standard CFRP production with a vacuum bag. Instead of an autoclave, a radiant heater is used to heat the part.

**Figure 1:**
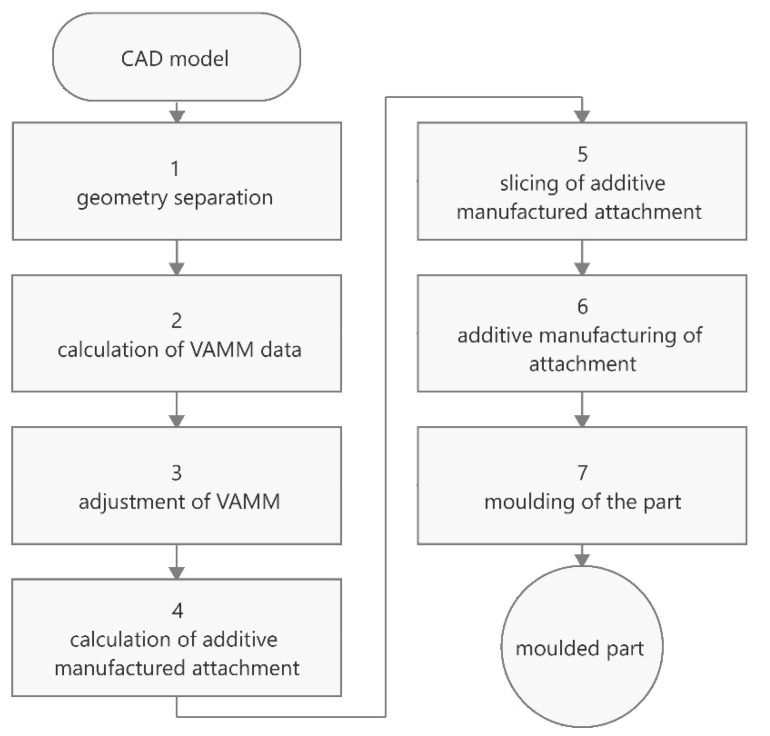
Process flow of the component manufacturing in the EMMA process (based on [1]).

In addition to the advantages of rapid mould setting and thus the rapid production of new components, this technology also has certain disadvantages. For example, the interpolation layer not only leads to a smoothing of the discrete height jumps between the pins but also to a smoothing of desired edges. This means that only features with comparatively large radii can be produced. Similarly, fine details cannot be manufactured with this technology, as the pins and the interpolation layer specify the minimum size of the details. To solve these problems and thus expand the geometries that can be represented, Lušić et al. [12] propose the use of additively manufactured attachments. These attachments represent the areas of the mould that cannot be set by the VAMM machine. In Herzog and Tille [1], the boundary conditions were analysed, and the process called enhanced multipoint moulding with additive attachments (EMMA) was defined. Figure 1 illustrates the workflow for EMMA.

The first step in EMMA is the geometry splitting based on the CAD model of the mould. The mould is divided into the part that can be represented by the VAMM machine and the part that requires an additively manufactured attachment. In the second step, the setting data for the VAMM machine are calculated, which include the heights of the individual pins on the one hand and the process parameters such as the vacuum pressure on the other. The pins of the VAMM machine are then set, the interpolation layer is stretched over the pin field and the machine vacuum is generated.

The calculation of the additively manufactured attachment is carried out subsequently or, if required, in parallel. The first step is to determine the exact geometry of the attachment. In this step, it must be ensured that the bottom geometry of the attachment corresponds to the geometry of the set interpolation layer at the location of the attachment. The slicing for the additive manufacturing of the attachment is then carried out with the exact path planning with the defined manufacturing parameters. The thereby generated G-code is then translated into a robot program for production and transferred to the robot. Once this has been transferred, the attachment can be manufactured.

The last step in EMMA is the fabrication of the actual component. For this purpose, the appropriate release agents are applied to the mould consisting of the VAMM machine and the additively manufactured attachment, which is then moulded with the selected material (e.g., CFRP). After applying the part vacuum and the corresponding curing time, the component can be demoulded, and the process ends with the moulded component. Further processing steps such as cutting the component to size can then follow.

After cleaning the mould consisting of the VAMM machine, the additively manufactured attachment is then available for further use. However, the basic requirement for the process is that the additively manufactured attachment can be used once. Therefore, it must be assumed that it has to be manufactured again for each moulding. This requirement follows from the primary orientation of the process towards single-part production, where it can be assumed that the next mould will require a different attachment in any case.

In this context, additive manufacturing on a silicone construction platform has already been investigated in previous studies [13,14]. Furthermore, there is already some research on material extrusion with six-axis robots in varying configurations and with different objectives [15,16,17,18,19,20,21,22]. However, no investigations are yet known to have been undertaken on the mouldability of the combined mould consisting of the VAMM machine and the additively manufactured attachment with CFRP. This novel aspect will be examined in this paper. Therefore, the positional accuracy of the attachments during moulding was investigated to ensure that the adhesion of the attachments to the silicone interpolation layer was sufficient. The accuracy of the manufactured components was also tested concurrently.

## 2. Materials and Methods

### 2.1. Experimental Setup

The experimental setup consists of the VAMM machine and the six-axis robot for extruder guidance. The VAMM machine was taken from the studies by Wimmer [10] and corresponds to the setup described there as a “full scale prototype”. This VAMM machine has a field of 572 pins in 52 rows, resulting in a usable moulding area of about 400 mm × 600 mm. The pins are of a hexagonal shape with a rounded head and are densely packed in the pin field. For the height adjustment, an internal trapezoidal threaded spindle is used, and the rows are adjusted sequentially.

**Figure 2:**
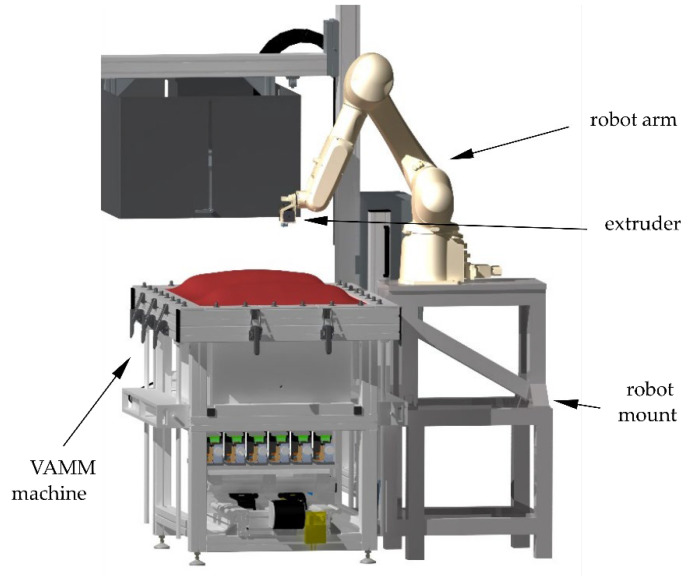
Rendering of the complete EMMA test setup [24].

For the additive manufacturing of the attachments, an extruder of type Titan Aero manufactured by E3D-Online Ltd., Chalgrove, Oxfordshire, UK, is used. This is guided by a six-axis industrial robot of the type RS007LFF60 made by Kawasaki Heavy Industries, Ltd. [23], Chūō, Kobe, Japan. The robot is mounted on a stable construction of aluminium profiles, which is assembled directly to the VAMM machine and thus creates a fixed connection. Figure 2 shows a rendering of the complete experimental setup. In addition to the system components described, the radiant heating of the VAMM machine, which is not used in these experiments, is also shown.

**Figure 3:**
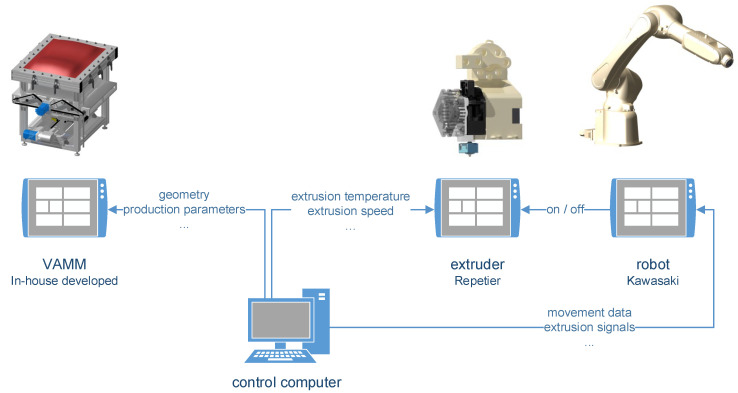
Schematic representation of the different controls of the individual components and their communication, consisting of the vacuum-assisted multipoint mould (VAMM) control, the extruder control and the robot control communicating via on/off signal.

To make it as easy as possible to separate the individual parts of the system, they are each equipped with separate controls. The interaction of these controls is shown in Figure 3. The VAMM machine relies on a control system specifically developed for the system which, like the entire system, was taken over from Wimmer [10]. An adapted version (based on version 1.0.0) of Repetier from Hot-World GmbH & Co. KG [25], Willich, Germany, was used to control the extruder. In this setup, the parameters extrusion temperature and extrusion speed are fixed due to the limited communication possibilities between the robot control and the extruder control. The extruder is activated and deactivated via an on/off signal from the robot control. The robot controller in turn corresponds to the controller supplied with the robot.

In addition to the easy separability of the individual system components and the associated easy reusability for other purposes, this control design also has certain disadvantages. On the one hand, there is the fixed extrusion speed, which also means that the travel speed of the robot cannot be selected variably. On the other hand, only a fixed extrusion temperature is possible, so an optimum between the individual component areas and the adhesion on the silicone platform had to be found. This determined combination of parameters with which the tests were carried out is explained in Section 2.2. together with the materials used.

**Figure 4:**
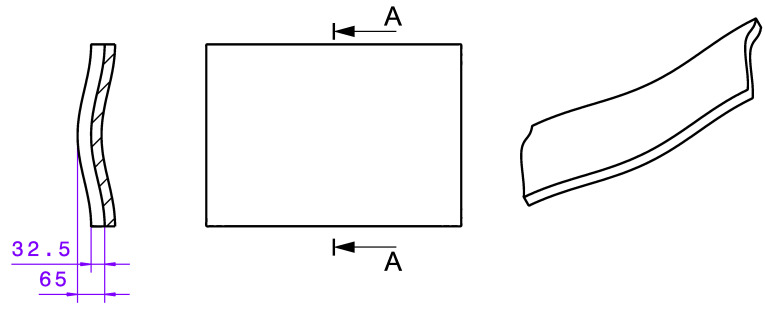
Dimensions and representation of the basic component shape represented with the VAMM machine. Unit: mm.

Two different geometries described below were defined for the tests carried out here. Both consist of a uniform base geometry, which can be set with the VAMM machine. This base geometry is composed of two superimposed cosine functions in the *x*- and *y*-directions and is reminiscent of a saddle in terms of its shape. The dimensions of this basic geometry are shown in Figure 4.

The height of the individual points of the base saddle geometry can be calculated with Formula (1). The formula is used to calculate the pin heights so that the thickness of the interpolation layer approximately results in an additional *z*-shift by the thickness of the interpolation layer. The formula characters *x*, *y* and *z* correspond to the respective coordinates in mm.

(1) $$z=\frac{\left(H-h\right)}{2}\times \left(\cos\left(\frac{2\cdot \Pi }{L}\times x\right)-\cos\left(\frac{2\cdot \Pi }{B}\times y\right)\right)+h$$

In Formula (1), the height constants *H* and *h* define the deflection of the saddle geometry. The constant H is the maximum height difference in the geometry and, according to Figure 4, it is 65 mm for the geometry used in this study. The constant h describes the maximum height difference for the short side and is therefore 32.5 mm. The constants *L* and *B* are the machine constants fitting the geometry to the pin field. In the case of the machine used in this study, they are *L* = 612 mm and *B* = 436.48 mm.

**Figure 5:**
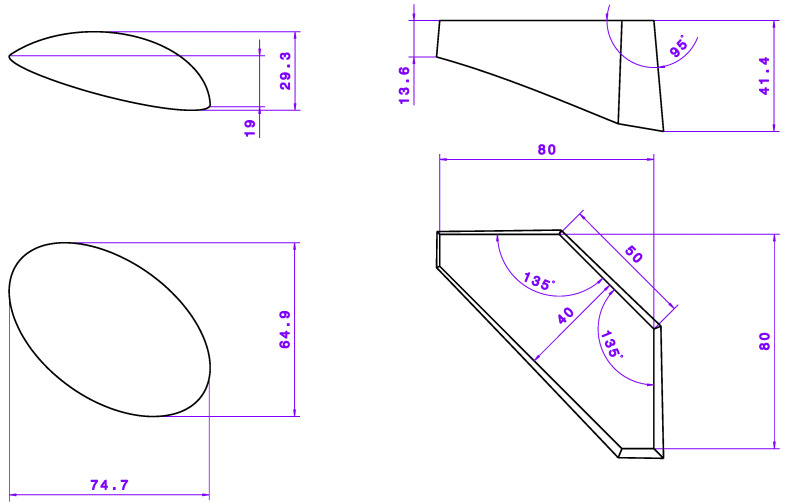
Dimensions of the additively manufactured attachments; specimen shape: round (**left**) and angular (**right**). Unit: mm.

In addition to the base geometry, two attachments with fundamentally different geometries were also defined. The base surface of the attachments is identical to the nominal surface of the base geometry, so that the contact surface of the additively manufactured attachments corresponds to the surface of the interpolation layer. One attachment geometry has a round shape, while the other is angular. The dimensions of the two attachments are shown in Figure 5. The two geometries represent two possible applications. The round geometry could, for example, represent a recessed grip, such as on the outer door handles of car doors. The angular geometry could represent a cable bushing or a reinforcement for a small window or control panel.

One specimen geometry is round with flowing shapes and slightly smaller. The other one is angular and sharp-edged in order to investigate the behaviour of the overall process. While in the case of the round geometry a relatively simple additive manufacturing with a low distortion can be assumed due to the rounding, a significantly stronger stair-step effect is to be expected with this geometry. Moreover, the round geometry can be expected to have relatively good mouldability with good separability of the attachment from the component at the same time. In contrast, the angular test specimen geometry can be expected to be more difficult to mould due to the only slightly rounded edges and the large height differences at the tip.

**Figure 6:**
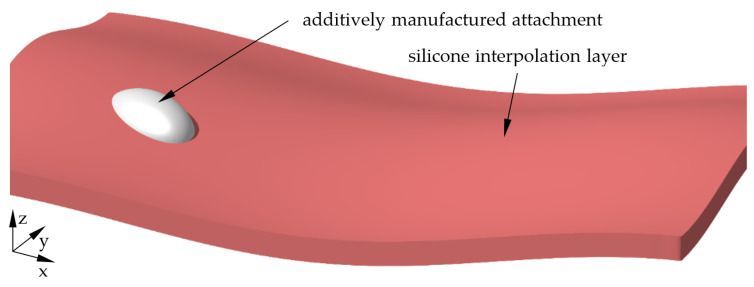
Overall shape of the mould for round specimen geometry.

**Figure 7:**
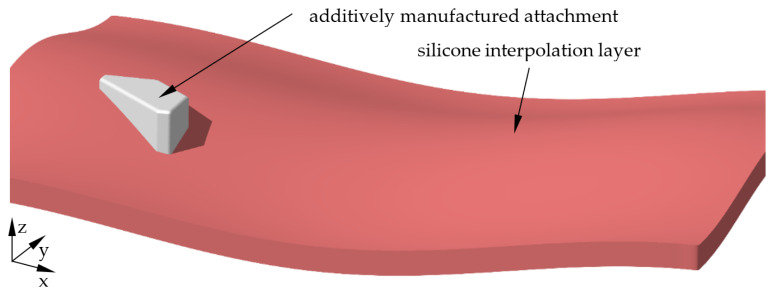
Overall shape of the mould for angular specimen geometry.

The total shapes of the mould for both geometries are shown in Figure 6 for the round geometry and Figure 7 for the angular geometry. Clearly visible in the rendering is the red interpolation layer in the saddle shape with the moulding attachments on top, which are shown in white.

The positioning of the attachments on the VAMM machine relates to a coordinate system with the origin in the centre of the VAMM machine. The coordinate directions are defined by an upward positive *z*-axis and a rightward positive *x*-axis along the longer edge, in accordance with Figure 6 and Figure 7. The coordinates to the centre of the attachments are shown in Table 1. The origin of the coordinate system in the *z*-direction is measured manually before the first attachment production.

The production of every specimen geometry was repeated three times for a definite statistical certainty of the results. The manufacturing of the test components on this assembled mould consisting of the VAMM machine and the additively manufactured attachment was carried out using the hand lay-up process with the materials described in more detail in the next section.

### 2.2. Materials

One of the challenges for the implementation of the EMMA is the adhesion of the additively manufactured attachments to the silicone interpolation layer of the VAMM machine. The silicone interpolation layer was taken from Wimmer [10], as was the rest of the system. The interpolation layer consists of red silicone mats of type VMQ with a hardness of 40 ShoreA from GaFa-Tec Handels GmbH [26], Schwielowsee, Germany. The entire interpolation layer consists of five mats with a thickness of 5 mm each, resulting in a total interpolation layer thickness of 25 mm. A layer of peel ply is placed between the silicone mats to allow the layers to slide over each other. While the top layer is led up to the tenter frame and takes over the vacuum sealing, the lower layers only cover the pin field itself to keep the forces acting on the tenter frame at a minimum.

The attachments were manufactured with white polylactide (PLA) from Verbatim GmbH [27], Eschborn, Germany. Adhesive stick Stick ecoLogo from Tesa SE [28], Norderstedt, Germany, was used as an adhesion promoter. The attachments were produced with a travel speed of 5 mm/s at an extrusion temperature of 220 °C. The layer thickness was 1 mm using a nozzle diameter of 1.5 mm.

For the tests carried out here, the mouldability is investigated with a hand lay-up process and a room temperature curing resin system. Following previous investigations, a carbon fibre twill fabric type HP-T195C from HP-Textiles GmbH [29], Schapen, Germany, was used. The resin system used was the corresponding epoxy resin system HP-E55L from HP-Textiles GmbH [30], Schapen, Germany. The moulding was made with a four-layer build-up of the above-mentioned carbon fibre fabric and the resin cured at room temperature. Curing took place under a vacuum bag at an absolute pressure of 50 mbar with a hysteresis of 200 mbar.

While the silicone interpolation layer itself has sufficient separating properties from the component and allows detachment, additional separating layers are necessary in the area of the additively manufactured attachments. For this purpose, the release wax type HP-G from HP-Textiles GmbH [31], Schapen, Germany, was used. In addition, a layer of cling film was added to prevent the resin from filling the pores of the additively manufactured attachments.

### 2.3. Evaluation Criteria

The major challenges in EMMA are the additive manufacturing of the attachments and the moulding of them. The aim of this article is to investigate the basic functionality of the entire EMMA process chain. In addition to the manufacturability of the attachments on the silicone building platform, which has already been investigated in previous work [13], the positional accuracy during moulding of these attachments must be demonstrated and the moulding result evaluated.

The evaluation of the positional accuracy of the attachments, i.e., the non-displacement during moulding due to sufficient adhesion on the silicone interpolation layer, is carried out with a comparison of two 3D scans. The first scan is performed after the additive manufacturing of the attachment and is used as the reference component. The second scan is carried out after the removal of the vacuum setup (vacuum bag, suction fleece, perforated foil, peel ply) with the fabricated component. The two scans are analysed with a surface comparison in GOM Inspect 2019 from Carl Zeiss GOM Metrology GmbH [32], Braunschweig, Germany. The result of this comparison is the representation of the offsets between before and after the moulding, so that a uniform offset in material thickness can be expected over the manufactured component.

In addition to the evaluation of the non-displacement of the attachments, the mouldability of the attachments must also be investigated. Therefore, the fabricated components are visually evaluated. The main focus is on the results in the area of the moulding attachments while the component quality itself is explicitly not to be evaluated. This means that any defects from the material application or the vacuum setup are not considered.

The third evaluation category serves to prove the dimensional accuracy of the components. For this purpose, target-actual comparisons are again used analogously to the first evaluation area. In contrast to the first evaluation, however, this evaluation does not refer to the scan before the moulding but to the target geometry of the mould from the CAD data. The evaluation should again refer to the area of the additively manufactured attachments. Therefore, a section around these is analysed.

## 3. Results and Discussion

### 3.1. Positional Accuracy of the Attachments

**Figure 8:**
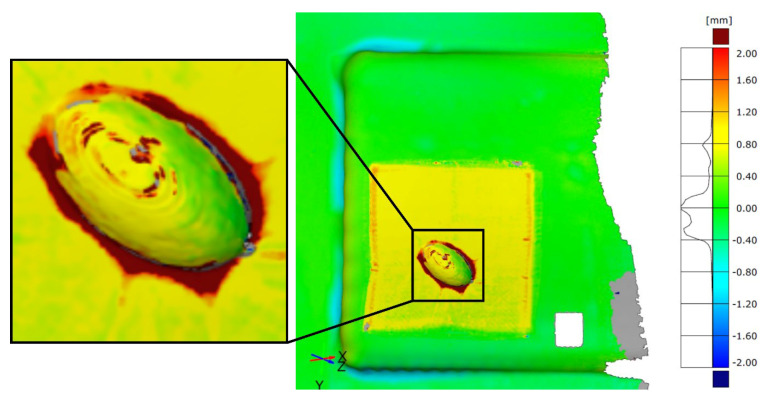
Surface comparison of the round test specimen geometry before and after moulding with a histogram of the deviations and enlarged section around the additively manufactured attachment (first test).

In accordance with the previously defined procedure, 3D scans before and after moulding are compared using a surface comparison to check the positional accuracy of the attachments. In the moulded area, a constant positive offset is to be expected, which should roughly correspond to the material thickness. For the round test specimen geometry and the first test, the comparison result is shown in Figure 8.

The area moulded with the CFRP can be clearly seen by a relatively even positive offset illustrated in yellow. From the histogram of the deviations, it can be seen that this offset is about 0.8 mm. This corresponds to about three times the thickness of the carbon fibre fabric of 0.3 mm and is thus somewhat below the expected fourfold material thickness. This is within the range that was to be expected due to the pressing of the fabric by the vacuum. The transition area between the additively manufactured attachment and the VAMM machine shows significantly larger deviations marked in red. In principle, this can be expected and explained by the moulding process. While the transition between the VAMM machine and the additively manufactured attachment is sharp-edged, the material thickness and the limited bending capacity of the moulding material lead to a rounding of the transition. The vacuum setup with peel ply, perforated foil, absorbent fleece and vacuum foil has a further influence. All these materials have only limited extensibility and therefore cannot compensate for the height differences in the mould by stretching. VDI 2014 Part 2 [33] prescribes a minimum radius of 5 mm for inner radii, which was not executed in the attachment but is approximately set by the moulding. At the same time, the excess material of these layers leads to a slight wrinkling on the upper side of the component, which is also visible.

In the area of the additively manufactured attachment, there is an offset mainly shown in yellow. One side also shows a transition into the green area, which suggests a slight displacement of the attachment during moulding. However, the opposite flat part of the component also shows a colouring thus indicating smaller offsets. This suggests that there is no displacement of the additively manufactured attachment relative to the silicone surface. Rather, the combination of the two differences indicates that the attachment was pressed into the silicone interpolation layer by the vacuum. Moreover, some influence could result from the mapping of the two scans in the best-fit process. Overall, this proves that the attachment was not displaced during moulding and remained in position.

**Figure 9:**
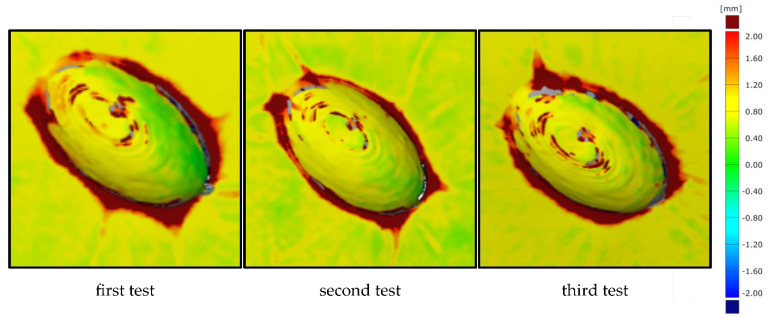
Surface comparison of the round test specimen geometry before and after moulding around the additively manufactured attachment for all three tests.

To better evaluate results, the tests were repeated three times and are compared in Figure 9.

Essentially, the second test shows the same results as the first test. The rounding out at the transition between the additively manufactured attachment and the interpolation layer is slightly less in this test. The previously explained correlations are again likely to be relevant as causes. It can also be clearly seen in this test that no significant shift of the additively manufactured attachment occurred. The surface comparison for the third test once again confirms the previously explained relationships. The relatively uniform colouring around the additively manufactured attachment shows that no significant displacement of the attachment can be detected here either. In general, a slightly lower application thickness between the scans can be seen on the upper sides of the attachments. This indicates that the component vacuum leads to a certain indentation of the additively manufactured attachment into the flexible silicone interpolation layer.

In general, the three repetitions for the round specimen geometry show very similar results with slight differences in the transition area between the additively manufactured attachment and the interpolation layer. These differences mainly result from the deviations caused by the manual draping of the individual layers in the hand lay-up process. Overall, all the results show a very uniform offset of the scans before and after component production, so that a displacement of the attachments during moulding can be rejected.

**Figure 10:**
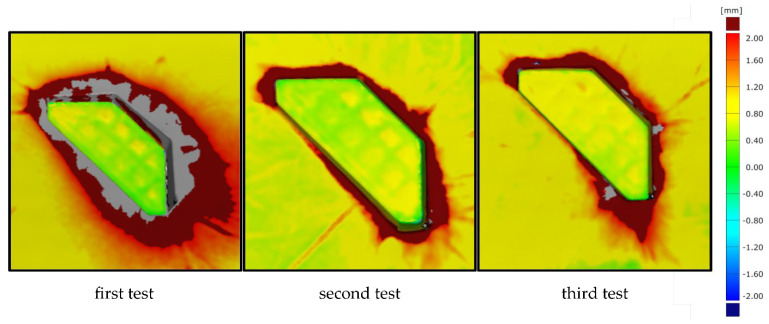
Surface comparison of the angular test specimen geometry before and after moulding around the additively manufactured attachment for all three tests.

In addition to the round specimen geometry, the angular specimen geometry is also investigated. The surface comparison for the three tests is shown in Figure 10. Compared to the round specimen geometries, it is noticeable that the angular specimen geometry is significantly more difficult to mould and shows greater deviations between the scans. There is clearly more pronounced filleting at the transition between the interpolation layer and the additively manufactured attachment. Although the additively manufactured attachment complies with the demoulding slope of at least 1°–2.5° specified in VDI 2014 Part 2 [33], the corresponding minimum rounding with a radius of 5 mm at the transition of the moulded parts was not produced due to the expected stair-step effect. However, the dark red colouring extending far outwards confirms a filleting clearly above this. The areas missing in the scan (grey) result from the glossy surface of the part, which is discussed in detail in Section 3.2. The surface comparison also shows the significantly stronger wrinkling in the CFRP component, which is again due to the correlations already described. The smaller-than-expected deviations on the upper side of the attachment suggest that the vacuum here presses the test specimen evenly into the silicone mat. At the same time, the even colouring of the edges of the attachment indicates that the additively manufactured moulding attachment was not displaced here either.

Compared to the first test, the second test shows significantly smaller deviations in the transition between the VAMM machine and the additively manufactured attachment. This lower rounding in the base of the additively manufactured attachments suggests a vacuum setup that follows the mould more closely. The shiny component areas in the transitions are also absent. The more difficult mouldability of the angular geometry in comparison with the round geometry is also confirmed. However, this test shows that the angular test specimen geometry can also be produced by EMMA without a displacement of the additive attachment occurring. The third test also shows slight deviations. The filleting at the mould transition is similar to the second test. They are most pronounced in the area of the greatest height difference at the front tip of the attachment. Moreover, for this test specimen geometry, no displacement of the attachment is recognisable.

An examination of all three tests shows that the results are significantly different from those of the round geometry. It is particularly noticeable that the transitions between the VAMM machine and the additively manufactured attachment are becoming better with increasing repetition. This shows the greater susceptibility to error of the moulding of the angular geometry due to the manual steps compared to the round test specimen geometry. At the same time, however, it was also possible to show that component manufacture is possible without displacement of the additively manufactured attachments with this geometry. This proves that the process can also be used for component manufacture in principle.

All in all, it could be shown that no displacement of the additively manufactured attachments occurs for both test specimen geometries with different moulding qualities. It is also clear that the moulding of the angular geometry with the selected combination of material and the rigid cover foil reaches its limits. The surface comparisons before and after the moulding thus show difficulties in certain areas when considering the entire mould. However, these are mainly due to the choice of material and the further process setup and not to the functional efficiency of the moulding with additively manufactured attachments, which is mainly being evaluated here.

In addition to the optical assessment, an evaluation should be made for the moulding tests on the basis of the permissible profile shape tolerances. According to ISO 20457:2018 [34], a tolerance of t = 4 mm is permissible for the moulded component. When considering the moulded area as a representative section for the entire mould, the permissible tolerance is t = 6 mm. This tolerance indicates the distance between two surfaces within which the component must be located. Thus, a tolerance of t = 4 mm means that either a deviation of ±2 mm is permissible or by parallel displacement in the range between 0 mm and 4 mm. When looking at the surface comparisons, it becomes clear that no negative form deviations occur in the area of the component. Therefore, a tolerance range of 0 to 4 mm applies.

The analysis of these surface comparisons shows that the maximum deviations occur in the transition between the additively manufactured attachment and the VAMM machine. For the round geometry, maximum values between 4.8 mm and 6.6 mm (depending on the test) can be measured. For the angular geometry, these maximum values are significantly higher at 7 mm to 8.7 mm. When subtracting the material thickness of 0.8 mm, the round test specimen geometry can meet the tolerance for the entire mould. In the case of the angular specimen geometries, this is not the case at the transition. Away from the transition, even the tolerances for the smaller part section can be met for all components produced.

This clearly shows that components can be produced within the permissible tolerances with the process if the appropriate design of the mould transitions is implemented.

### 3.2. Mouldability of the Attachments

In addition to the proof of non-displacement, the second criterion is the visual evaluation of the mouldability of the entire mould. For this purpose, individual test results are discussed in this section as examples.

**Figure 11:**
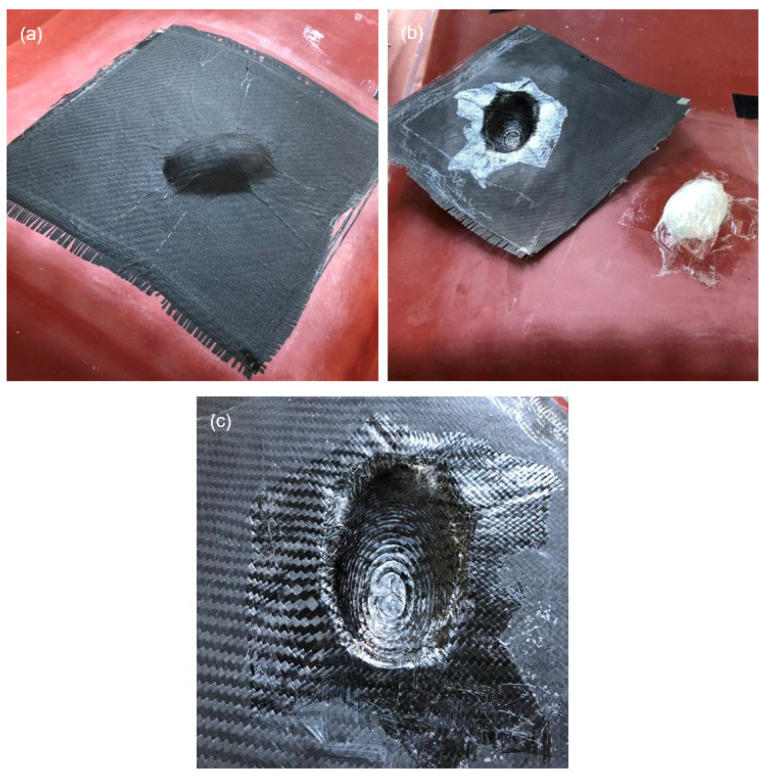
Moulding result of the round test specimen geometry (exemplary first test) (**a**) after removal of the vacuum bag, (**b**) after detachment of the CFRP component and (**c**) CFRP component in the area of the additively manufactured attachment.

Analogous to the previous subsection, the evaluation of the moulding result is also to be carried out here first for the round specimen geometry. Therefore, the CFRP component is shown in different demoulding stages in Figure 11. After removal of the vacuum setup, a matt surface of the component can be seen in Figure 11a, as expected. The attachment is formed in detail, with the already-described rounding in the transition between the attachment and the interpolation layer. Also visible is a slight wrinkling in the material, which is essentially due to the further vacuum setup, as already discussed. The relatively low drapeability of the peel ply used is responsible for most of the wrinkles. Moreover, the vacuum bag is not stretchable; therefore, additional film must be draped to achieve a uniform vacuum pressure and thus a good moulding of the transition between the mould parts. This excess material subsequently leads to additional wrinkles in the other part areas.

The retention of the additively manufactured attachment on the silicone interpolation layer after demoulding as shown in Figure 11b shows a level of performance that goes beyond the originally set goals. The aim was a single moulding of the additively manufactured attachment. However, due to the high adhesion that can be achieved, multiple mouldings can also be considered for this test specimen geometry.

The bottom of the component shown in Figure 11c in turn confirms the overall positive moulding result of the upper side of the component, and almost no wrinkling can be seen. While the carbon fibres on the upper side of the component smooth out the stair-step effect of the additively manufactured attachment, this is reproduced on the bottom by the resin flowing in. It can be assumed that there is no impairment of the component’s functionality caused by this effect. The somewhat uneven radius formation in the rounding is also as expected as it is not represented by the attachment. Overall, the objectives for this test specimen geometry can be regarded as fulfilled.

**Figure 12:**
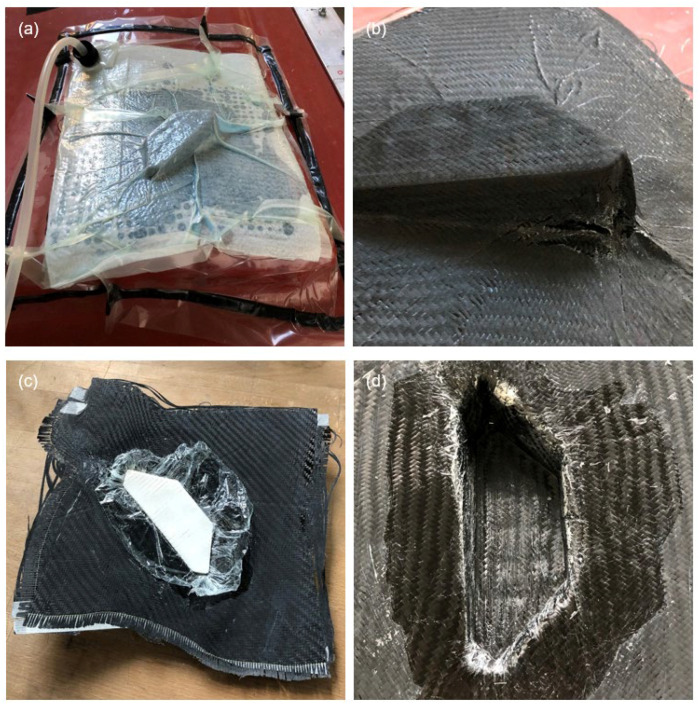
Moulding result of the angular test specimen geometry (exemplary third test) (**a**) with vacuum build-up, (**b**) after removal of the vacuum bag, (**c**) after detachment of the CFRP component and (**d**) CFRP component in the area of the additively manufactured attachment.

Figure 12 shows the moulding for the angular specimen geometry. Figure 12a shows the aforementioned wrinkling in the upper material of the vacuum build-up. These wrinkles are partially transferred to the component surface. This transfer is visible in Figure 12b, which also shows additional wrinkles due to the exceeded compensation capacity of the carbon fibre fabric. The shiny areas in the transition between the VAMM machine and the additively manufactured attachment described in the previous section can also be seen. These occur when the peel ply does not attach to the material and the resin can thus form a smooth and shiny surface. In principle, the defects shown are mainly optical defects, which should not lead to any impairment of usability. For a highly stressed component, a fibre structure calculated in accordance with the stresses and thus significantly more complicated would have to be used according to VDI 2014 Part 3 [35] and would also avoid these effects.

Figure 12c shows that multiple mouldings cannot be achieved for this specimen geometry. The foil allows the attachment to be removed from the component without any problems. According to the objective, this also completely fulfils the requirement of a single moulding. In Figure 12d, analogous to the previous test specimen geometry, it can be seen that the wrinkling on the bottom of the component is significantly less. Only a slight waviness from the folds on the other side is present. At the same time, the fillet with different radii in the transition area of the interpolation layer and the additively manufactured attachment show that a representation of the radius specified according to VDI 2014 Part 2 [33] in the transition of the mould parts would be preferable.

With regard to the principle mouldability of the attachments to be investigated here, this could also be shown for this specimen geometry. It is also clear that the moulding of the angular geometry places increased demands on the processing. At the same time, the angular transitions, the low demoulding slope and the high-rising corner also cause deviations in the further layer structure. The changes in shape also lead to wrinkling in the peel ply, the absorbent fleece, the perforated film and the vacuum film, which is then transferred to the component.

Overall, the production results and the surface comparisons show that EMMA in the presented stage meets the goals set. There is potential for improvement in the moulding quality. At the same time, it is to be expected that a form-oriented specification of the rounding radii in the transition of the mould parts will lead to improved moulding quality.

### 3.3. Accordance to Nominal Geometry

The third evaluation criterion is the conformity of the moulded part with the target geometry. The evaluation should refer to the area around the additively manufactured attachment, which is the focus of this research project. Therefore, a surface comparison is carried out between the CAD model of the mould and the surface scan of the moulded component. Typical representatives are discussed in this section as examples.

**Figure 13:**
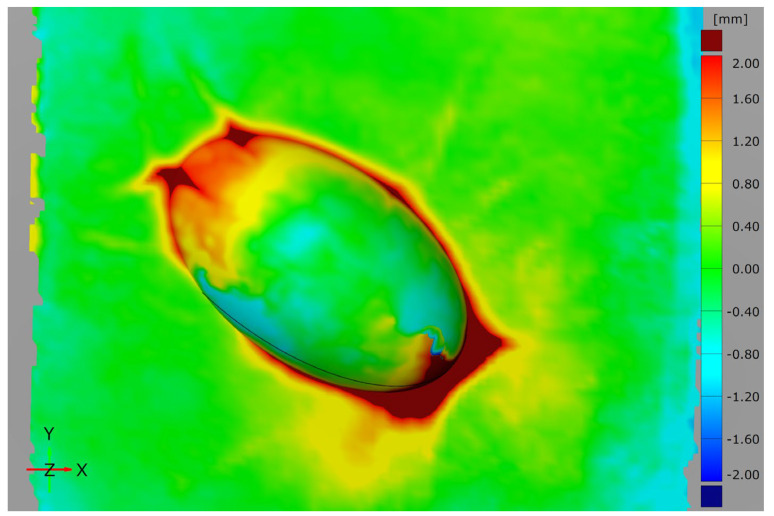
Comparison to the nominal geometry in the area of the additively manufactured attachment for the round test specimen (second test).

Figure 13 shows this surface comparison for the second test with the round geometry. It becomes clear that there is relatively good agreement with the target geometry overall. The deviations are again clearly visible in the area of the transition between the attachment and the basic geometry. Since the nominal geometry in this comparison refers to the mould, an offset in the material thickness is again to be expected here. However, this only occurs in the lateral areas of the attachment. The constant offset in the height direction (viewing direction) is omitted, as the comparison is made using a best-fit algorithm; thus, the height offset in this direction can be compensated. Slight positive deviations can be seen in the upper area. It is conceivable that the material is slightly less compressed during the manufacturing process or that the material is thickened due to a lower stretching of the fabric in this area. The slight deviations at the edge of the cut-out suggest a slight bulge in the interpolation layer in the area of the attachment, which also contributes to this.

In contrast to the comparison shown in Figure 9, it is noticeable that the deviations at the attachment tips do not occur there. A comparison of the scan data before moulding and the nominal geometry confirms that the deviations already existed before moulding. Possible causes are deviations in the production process of the attachment. For example, it is possible that there is too little material being applied in the area of the higher part of the attachment and an over-application of material in the lower attachment area. At the same time, there is also the possibility that the material remains deformable for too long due to the comparatively large amounts of material applied in the thicker areas. Thus, the structure slightly collapses during production. Another explanation for this behaviour could also be a non-detected deviation in the silicone interpolation layer, which leads to an overall virtual shift of the scans when matching by the best-fit algorithm.

**Figure 14:**
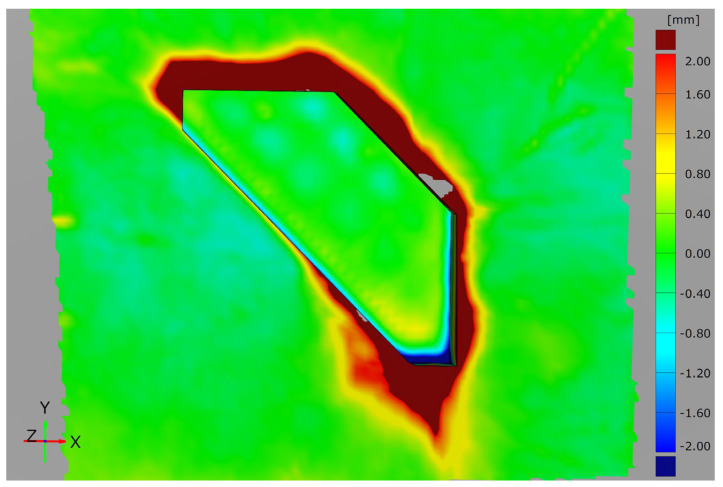
Comparison to the nominal geometry in the area of the additively manufactured attachment for the angular test specimen (third test).

Figure 14 shows the comparison for the third test with the angular specimen. There is also a bigger deviation in the area of the thicker part of the attachment. It is clearly visible that the material thins out considerably at the front corner and the sharp edges. This thinning is due to the sharp transitions of the edges with the corresponding stretching of the material. A further factor is the pressure applied to this area by the vacuum build-up. In addition, this thinning at the edge also leads to the visible slight thickening of the material.

Overall, the surface comparison for this specimen geometry yields the greater deviations, as already expected from the previous evaluations. In addition to the deviations in the upper area, these are also primarily the large deviations in the transition area of the VAMM machine and the additively manufactured attachment.

When looking at the entire evaluation for this criterion, it becomes clear that the moulding accuracy in the area of the attachments is relatively high. Deviations occur mainly in the area of the transitions between the additively manufactured attachment and the interpolation layer, whose design should also include a transition radius according to VDI 2014 Part 2 [33]. As a result, this evaluation also leads to an overestimation of these areas in comparison to the standard-compliant design. A more precise and detailed investigation of the exact reasons for the deviations requires further work, which would go beyond the scope of this paper.

## 4. Summary and Outlook

The aim of these investigations was to prove the mouldability of the additively manufactured attachments in enhanced multipoint moulding with additive attachments. Two specimen geometries were defined and manufactured using enhanced multipoint moulding with additive attachments. The manufactured test specimens were then further analysed. On the one hand, a surface comparison was carried out to evaluate if the attachments shift during production. The second evaluation criterion was a visual evaluation of the test specimens. The third part of the evaluation refers to the surface comparison of the components with the nominal geometries.

The investigation of the non-displacement of the additively manufactured attachments during impression taking showed sufficient adhesion of the attachments to the silicone interpolation layer for both test specimen geometries. Due to this sufficient adhesion, no measurable displacement of the attachments occurred, and the principal usability of the process could be demonstrated. On the other hand, this evaluation also shows certain deviations of the components from the target geometry in the transition area of the VAMM machine to the additively manufactured attachments. These deviations are more pronounced in the case of the angular test piece geometry and can be attributed on the one hand to the geometry and the vacuum build-up. On the other hand, however, the rounding radii in the transition area that were not implemented in the test should also be mentioned.

The visual evaluation of the test results shows a good component quality with only slight wrinkling on the surface, which is due to the vacuum build-up. These are again more pronounced in the angular test specimen geometry and the transition area between the interpolation layer and the additively manufactured attachment. However, this evaluation also shows good component quality.

The comparison of the 3D scans with the target geometry also shows a relatively good agreement but also certain deviations, especially in the higher areas of the attachments. However, the exact reasons for this need further investigation. This investigation also shows that, with the exception of the transition areas between the additively manufactured attachment and the interpolation layer, the shape deviations permissible according to the standard ISO 20457:2018 [34] can be met.

Based on these findings, further investigations should focus on the design of the transition radii between the additively manufactured attachments and the VAMM machine. In addition to the constructive improvement of the transitions, the investigation of the improvement of the impression through a possible change of the vacuum structure also comes into play. At the same time, future research offers the possibility to further increase the moulding quality and to reduce the production times, thus making the process even more efficient.

The novelty of this study is the demonstration that enhanced multipoint moulding with additive attachments is usable for production of CFRP parts at the current stage and achieves the defined goals. At the same time, this process significantly expands the range of adjustable geometries. Therefore, in the future, this process can certainly make a contribution to resource saving and more efficient and faster production of individual parts and small series.

## Figures and Tables

**Table 1:** Coordinates of the attachment centre relative to the centre of the vacuum-assisted multipoint mould. Unit: mm.

	x-Coordinate	y-Coordinate	z-Coordinate
Round specimen geometry	−181.0	−73.5	28.1
Angular specimen geometry	−191.0	−78.5	23.6

## Data Availability

The raw data required to reproduce the findings of this published article are available from the corresponding author upon reasonable request.
